# Private sector malaria RDT initiative in Nigeria: lessons from an end-of-project stakeholder engagement meeting

**DOI:** 10.1186/s12936-018-2222-8

**Published:** 2018-02-06

**Authors:** Babatunde Odugbemi, Chijioke Ezeudu, Anyiekere Ekanem, Maxwell Kolawole, Idowu Akanmu, Aderemi Olawole, Nkabono Nglass, Chinwe Nze, Edward Idenu, Bala Mohammed Audu, Godwin Ntadom, Wondimagegnehu Alemu, Rex Mpazanje, Jane Cunningham, Augustine Akubue, Tolu Arowolo, Seye Babatunde

**Affiliations:** 1 Department of Community Health and Primary Health Care, Lagos State University Teaching Hospital, Lagos, Nigeria; 2 Department of Paediatrics, Nnamdi Azikwe University, Awka, Nigeria; 3 Department of Community Health, University of Uyo, Uyo, Nigeria; 4 Malaria Consortium, Abuja, Nigeria; 5 National Malaria Elimination Programme, Federal Ministry of Health, Abuja, Nigeria; 6 World Health Organization, UN House, Plot 617/618, Central Area District, Garki, Abuja, Nigeria; 7 Global Malaria Programme, Geneva, Switzerland

**Keywords:** Malaria, Rapid diagnostic test, Private providers, Stakeholder engagement, Nigeria

## Abstract

The malaria rapid diagnosis testing (RDT) landscape is rapidly evolving in health care delivery in Nigeria with many stakeholders playing or having potential for critical roles. A recent UNITAID grant supported a pilot project on the deployment of quality-assured RDTs among formal and informal private service outlets in three states in Nigeria. This paper describes findings from a series of stakeholder engagement meetings held at the conclusion of the project. The agreed meeting structure was a combination of plenary presentations, structured facilitated discussions, and nominal group techniques to achieve consensus. Rapporteurs recorded the meeting proceeding and summaries of the major areas of discussion and consensus points through a retrospective thematic analysis of the submitted meeting reports. Key findings indicate that private providers were confident in the use of RDTs for malaria diagnosis and believed it has improved the quality of their services. However, concerns were raised about continued access to quality-assured RDT kits. Going forward, stakeholders recommended increasing client-driven demand, and continuous training and supervision of providers through integration with existing monitoring and supervision mechanisms.

## Background

Malaria remains a major cause of morbidity and mortality in Nigeria. The 2015 World Malaria Report showed that the global burden of malaria morbidity and mortality is dominated by Nigeria, accounting for about 29% of the global malaria disease burden [1]. Most fever cases in Nigeria are considered as malaria and treated as such without laboratory confirmation [2, 3]. Therefore, improving and scaling up laboratory diagnosis to ensure confirmation of malaria cases before treatment is one of the strategic thrusts of the National Malaria Elimination Programme [4, 5]. Thus, since 2010, Nigeria updated its National Guidelines for Diagnosis and Treatment for Malaria in line with the World Health Organization (WHO) recommendations that all suspected malaria cases must be confirmed by parasitological confirmation either by malaria rapid diagnostic test (RDT) or by malaria microscopy [4].

However, despite wide dissemination of the guidelines, continued prescription of anti-malarial medicines based solely on clinical suspicion remains a major practice in fever case management among health providers [6, 7]. This practice is more prevalent in the private health sector where more than 60% of all fever cases are seen [3, 8].

Evidence from surveys involving households and health care providers have consistently showed that many patients do not receive treatment according to the recommended guidelines at health facilities [3, 7–11]. Also, care seekers are most likely to patronize the private sector outlets especially the informal providers often referred to as proprietary patent medicine vendors (PPMVs) in Nigeria [3, 8]. The major reason why care seekers choose to patronize chemists, pharmacies, shops and even illegal drug sellers is the relative ease of purchasing drugs and obtaining immediate treatment [12].

The extension and promotion of the use of RDTs into the formal and informal private sector health system by the WHO was, therefore, aptly aimed at improving the quality of care and universal access to malaria diagnostic testing [13]. RDTs use requires a short training period, little expertise, does not require electricity, and provides a good opportunity for improved fever case management especially at lower levels of the health system and the vibrant and expanding private sector, where a high percentage of patients are seen [13].

An innovative, multi-partner collaborative project was recently piloted in three of the states in Nigeria [14] aimed at increasing the availability of affordable quality-assured RDTs in both the formal and informal private sector using a commodity market-stimulation approach. The project was implemented in three out of the 37 states in Nigeria, namely Ogun, Anambra and Cross River States. Each one had been purposively selected from the Southern three of the existing six geo-political zones of the country. Within each state, three local government areas were in turn designated as project areas. These local government areas were Aguata, Awka South and Idemili North in Anambra State; Calabar Municipality, Ikom and Obudu in Cross River State; Ado-Odo/Ota, Ifo and Sagamu in Ogun State.

As a new project, stakeholders’ engagement was identified as critical to the success of the project at all its stages including design, implementation, monitoring and evaluation, and reporting [15]. It was also recognized that creating avenues for stakeholders to ‘air’ own insights and perspectives is pertinent to ensuring the ownership and sustainability of project achievements and impact during and beyond the lifetime of the project. Thus, in addition to earlier engagements in the course of the project, a stakeholders’ meeting was organized as part of the end-of-project activities in each of the three states. The main objective of the meeting was to share project achievements, products, challenges and lessons in a bid to develop and inform potential plans for the expansion of RDT use for fever case management among private health providers and sustaining project gains in the states.

The aim of this paper is to summarize and disseminate the proceedings and outcomes of an end-process stakeholders’ engagement meeting at the conclusion of a pilot project on the deployment and use of RDTs among private sector health providers in three states in Nigeria.

## Methods

### Participants

The approach used for the end-of-project stakeholders meeting were plenary presentations, facilitated discussions, nominal group technique and thematic analysis.

Three separate 1-day meetings were convened at each of the project state on 19th, 21st and 26th April 2016 by the World Health Organization and the UK/DFID Malaria Consortium, the project’s main implementing partners, with funding from a UNITAID grant. A reference list of stakeholders, that had been identified and used at earlier stages of the project, was updated for contact details, and served as a check-list for invitations sent by email and/or telephone SMS. In addition, voice calls were made as a follow-up for those that an acknowledgement of the initial invitation was not received. Invitation letters were made on government letterhead and signed by the Programme Managers of the State Malaria Elimination Programme of the Ministry of Health. The letters were then scanned and sent electronically through the WHO email.

There were a total of 140 meeting attendees from across three categories of stakeholder types, shown in Table 1. Primary stakeholders were essentially the target audience of private providers who had participated in the project as operators or proprietors of privately-run service outlets, comprising private clinics, pharmacy shops and patent medicine shops (i.e. PPMVs). Invited representatives of the professional associations, through which the participants were originally recruited for the project, were also categorized under ‘primary’ stakeholders. Secondary stakeholders were taken to be representatives of the project-implementing partners with the addition of other development partners involved in malaria RDT interventions within the project states. National and State-level agencies of government whose activities involve the regulation of personnel, programmes or products that are related to malaria diagnosis and treatment were categorized as tertiary stakeholders. The other category of stakeholders included participants from the research institutes or universities within the project area, and also members of the press.

**Table 1:** Analysis of stakeholders at the end-of-project meetings at three sites

Stakeholder type	Stakeholder
Primary	Association of General and Private Medical Practitioners of Nigeria (AGPMPN), National Association of Proprietary Patent Medicine Dealers (NAPPMED), Pharmacists Council of Nigeria (PCN)
Secondary	WHO, Malaria Consortium, Society for Family Health (SFH), Clinton Health Access Initiative (CHAI), and UNITAID
Tertiary	Regulatory agencies including the States Ministry of Health, States Malaria Elimination Programme (SMEP), National Malaria Elimination Programme (NMEP), and National Agency for Food and Drug Administration and Control (NAFDAC)
Others	Members of the university community and academia with expertise in fields including clinical care, laboratory science, and public health; and members of the press

### Meeting agenda

Prior to the meetings, four members of the Project Coordination Team (PCT) from WHO and Malaria Consortium had met to develop, revise and finalize drafts of the meeting agenda. Based on the agenda, the PCT members also prepared presentations on topical issues that was agreed would serve as appropriate ‘triggers’ to prompt participants to share experiences and suggest solutions for sustainability and scale-up plans. The topics were Overview of the UNITAID RDT Project; WHO Recommendations on Malaria Diagnosis; and the current National Malaria Treatment and Diagnosis Guidelines. Thus, the agreed meeting structure was a combination of plenary presentations/discussion, and nominal group techniques to achieve consensus [16, 17].

The first round of discussion, facilitated by a PCT member, was held during plenary after the key presentations. It was directed to specifically focus on clarification of information, additional insights, and identification of concerns and perspectives on the completed project. The second round of discussion was more in-depth and conducted in syndicate groups during which the nominal technique was employed to reach a consensus among stakeholders on future actions to enhance sustainability and scale-up of RDT use in the private sector. Using Delphi Technique, the PCT members had developed a pre-meeting Topic/Discussion Guide, based on a retrospective thematic analysis of the concluded project outcomes and future expectations. The four identified topics, and thus discussion groups, were operational challenges; supervision, reporting and [service] quality assurance; market sustainability; and capacity building. For the discussions within each group, six variables guided their outputs: emergent issues; recommended actions; responsible action party; required resources; timeline; and means of verifying action taken.

All the participants were assigned by systematic random selection into the four discussion groups. Each group was instructed to appoint a Chairperson, note-taker, and presenter to guide and provide feedback at a second plenary session that followed. The feedback and second plenary was facilitated by a PCT member, and it focused mainly on achieving consensus on the group submissions.

### Meeting report logistics

Each meeting at the three sites was attended by a university academic that had been pre-selected and invited by the PCT members, based on a set of criteria. These included being a young academic (2–5 years post-qualification), research interest in malaria, previous publishing experience, proximity to project/meeting site, availability on meeting date, willingness to serve as a meeting rapporteur, and willingness to submit a meeting report within 1 week of the exercise. The university academics and the PCT members convened 2 months after the meetings at a 4-day retreat to summarize the submitted meeting reports. A draft manuscript was developed by summarizing the major areas of discussion and consensus points through a retrospective thematic analysis of the submitted meeting reports. This was further developed over several weeks by all authors by correspondence via emails.

## Results

The outcomes of the discussions and consensus reached by the 140 participating stakeholders are outlined below and summarized in Table 2. The findings are presented under the four *pre*-*defined themes*, but also categorized under items that were consequently identified as *emergent sub*-*themes*. By consensus, any reference to future actions for the “project implementers”, that is the development partners that coordinated the concluded project and the meeting, was taken as actions for “development partners” in general.

**Table 2:** Action plan for building sustainable market from group work and plenary discussions

Issues	Recommendations	Person responsible
Operational issues		
Relative low consumer demand for quality-assured malaria RDTs	Strategic behavioural change communication and marketing campaigns for RDTs Branding of participating service outlets	NMEP/SMEP Manufacturers Distributors Partners
Slow stock depletion	Provision of incentives for RDT use to service providers and clients Branding of competing products meant for public sector with ‘NOT FOR SALE’	NMEP/SMEP Manufacturers Distributors Service providers
Delayed supply of mRDTs by distributors	Decentralization by providing storage/sales hubs at each local government area	Distributors
Non-adherence to SOP	Closer supervision of care providers including refresher trainings and provision of job aids	NMEP SMEP Partners Professional groups
Drying-up of buffer solution	RDT kits to be checked at point of use and faulty kits should be reported to the manufacturer Supply of visual aids with kits	Manufacturers Distributors Service providers
Insistence on ACT by clients with negative results	Proper counseling of clients	Service providers
Supervision, reporting and quality assurance		
Limitation of data collection and reporting systems	Strengthening of data collection system by training and provision of standard reporting tools	NMEP/SMEP Partners Professional groups
	Submission of data to relevant government institutions and systems	Service providers
Inadequate monitoring of service provision	Build capacity of LGA malaria focal persons to provide additional supportive supervision and monitoring	NMEP/SMEP Partners
Absence of periodic Performance Reviews (on pricing, sales, end user perception, quality, innovations and best practices)	Institute periodic performance review meetings	NMEP/SMEP Partners Professional groups Distributors
Market sustainability		
Provider and client confidence on test results	Ensure only quality assured products are available to trained users	Manufacturers Regulatory agencies e.g. NAFDAC
Marketer-client relations	Training of marketers on interpersonal communication	Marketers clients
Leakage of public sector RDTs into the private open market	Introduction of price control to make RDTs affordable for consumers and profitable for providers	Government agencies WHO
Capacity building		
Knowledge gaps among service providers	Ongoing training/retraining of providers (especially health workers)	SMEP Partners
	Trainings should be more detailed and tailored to audience (conveyed in simple language)	SMEP Partners
	Trainings should be delivered in local languages	SMEP Partners
Limited number of participating private providers	More PPMVs and private doctors should be trained	SMEP Partners Professional associations
Limited number of qualified RDT trainers	Training and accreditation of additional trainers	NMEP Professional regulatory agencies

### Operational issues

#### Consumer demand

Participants reported that the demand for RDT testing by clients was low at the start of the concluded project, but that a gradual improvement was witnessed due to the increase in the awareness campaigns conducted by the project implementers. Those that participated as service providers mentioned their personal attempts in actively promoting RDTs when clients visited with only the intention to buy anti-malarial drugs. To ensure scale-up and continued use of RDTs, participants suggested more public campaigns through the use of diverse methods, including airing of jingles on popular radio and television stations. One of the participants offered an enthusiastic response to the increasing consumer-driven demand for RDT:

*“It is genuine and people love it, it can be sustained”.* (Private health worker/PPMV)

#### Stock depletion

Participants attested to a slow but gradual use of RDTs in the private sector, which in addition to the wrong approach to stock management by some providers, resulted in low stock depletion and high retention of stocks of RDTs that were close to expiration. This accounted for the opinion voiced by one participant:

*“Some may have been tempted to use expired test kits to limit their losses”.* (Private health worker/PPMV)

Categorization of the suggested future actions and responsible parties showed that they were similar in themes to those mentioned against ‘consumer demand’. There were additional comments and suggestions that low stock depletion resulted from the use of cheaper alternative kits that leaked from the public sector. These were mainly by participants representing development partners, marketers and regulatory agencies:

*“Partners and wholesalers should request manufacturers to clearly label donated products as “not for sale” to differentiate them from profit*-*driven products meant to be sold in the private sector.”* (Regulatory agency)

#### Supply of commodities

Participants also alluded to reports of frequent delays in the supply of RDTs by the marketers/distributors. During the project, RDTs were distributed from central locations within the project states and at specified time intervals, which sometimes resulted in erratic RDT supply to outlets farther away from the central supply points. Distributors were encouraged to decentralize by establishing “hubs” at lower levels that are closer to the service outlets, for instance at every local government area (LGA) headquarters.

#### Non-adherence to SOPs

The participants that were service providers in the concluded project admitted that training and materials were given to them on guidelines/standard operating procedures (SOPs) on use of RDTs. However, they reported that “many” providers did not adhere to the instructions that antimalarial drugs should be offered only when the tests are positive. This was perceived as an ‘operational’ issue although the corresponding actions that were suggested centered on providing closer supervision and re-training of service providers.

*“We use RDTs in my facility but even if the result is negative clinicians still prescribe anti*-*malarial drugs”.* (Health worker, State hospital)

#### Drying-up of buffer solution

To contextualize this finding, it must be mentioned that the concluded project deployed a customized single-use devise/buffer kit in preference to the multi-use test kits where buffer is supplied in a larger volume vial [18]. Some providers shared their experiences of shortages in the quantity of the buffer fluid in the test kit. They mentioned that the cause of the shortage was unclear to them, but speculated that it was from the point of manufacture or drying up of the liquid during storage. They noted that in some instances providers resorted to the use of other buffer alternatives rather than discarding the test kits, which may have rendered some results invalid.

*“If I carry out test with [named brand]… I find that the buffer is not sufficient”.* (PPMV)

They recommended that manufacturers and distributors should conduct proper inspection of products, and rectify the faults before supply and distribution to the service providers. However, during the discussions, there was a quick update on the WHO guidance that visual aids and training on their use should accompany the supply of such special kits [17], thus the participants recommended that action for the future.

#### Preference by clients

Although a significant number of clients accepted RDT testing, providers reported that there were varying reactions to negative results by their clients; some did not accept a negative RDT test result and insisted on purchasing artemisinin-based combination therapy (ACT) medicines. This particular issue prompted more discussions that related to provider-behaviour in terms of adherence to and trust in RDT results, which were categorized under other themes. The specific recommended action was that providers should spend more time in counseling clients.

### Supervision, reporting and quality assurance

#### Data reporting systems

Comparison was made between the initial and more traditional use of a paper-based data collection and monitoring system that was used during the concluded project, and a later migration to an electronic mobile devise-based system to monitor RDT and ACT use. The participants surmised that the paper-based system was tedious and limited their effectiveness in service provision; while the electronic system was also beset with network connectivity problems and software errors. The consensus on the way forward was to strengthen the system and conduct more training for providers to use the tools more efficiently.

#### Monitoring of service quality

The deficiencies in the collection and reporting of service data were linked with inadequate monitoring and supervision of service providers. Stakeholders from the government in particular pointed out that both data monitoring and supervision of private providers were not integrated into the existing established systems. In keeping with this, they recommended that the capacity of designated focal persons for M&E and supportive supervision at the local government/district levels should be built to enable them extend coverage to the private sector.

#### Performance review

Participants observed that there was no forum to address concerns and prompt feedback for improving the performance of private sector providers and the quality of service. Stakeholders agreed on a need for a periodic performance review meetings or other fora that could enhance an interaction of providers and other stakeholders.

### Ensuring scale up and sustainability of RDT markets

#### Provider and client confidence on test results

It was observed that the introduction of RDTs improved the profit margin for PPMVs due to the extra income from charging for the test. However, many of the providers expressed concerns about how to deal with situations in which test results conflict between RDTs and microscopy. This had resulted in many to doubt the validity of RDT results.

*“RDTs have resulted in rapid reduction in malaria but my fear is with quality. Multiple manufacturers and brands in the market. A particular one in my facility gave negative RDTs but was positive on microscopy”.* (Private medical practitioner)

*“Sometimes I get negative results, and then the patient goes to the lab for microscopy and comes back with a result with [malaria] “plus*–*plus*–*plus”. This leads to argument and doubt of the RDT…”* (PPMV)

The initial plenary presentations at the meeting had provided the participants with information to reinforce the evidence for the efficacy of RDTs, thus, though this issue generated a long and keen discussion, they were able to reach the consensus that only quality-assured products should be made available and to trained providers. This was seen as a means to ensure that both providers and clients will be confident to continue with the use of RDTs.

#### Marketer-provider relations

It was reported that poor communication between some providers and marketers resulted in a ‘break’ in the supply chain. It was perceived as the reason why some service providers in the concluded project, particularly those located far from RDTs marketers’ distribution points, pulled out of the project. Providers at the meeting expressed a desire to work closer with the marketers. There was consensus that marketers and providers must maintain a close relationship to ensure timely and continuous supply, and utilization of RDTs.

#### Leakage of public sector RDTs into the private open market

A lengthy discussion was held on the observations that donor-subsidized RDTs had leaked into the open market and thus resulted in the low stock depletion experience during the concluded project. Marketers and development partners also expressed concerns over the proliferation of a variety of non-quality assured RDTs in the open market. Consequently, service providers indicated that they had little knowledge on how to identify good quality RDTs, and requested for more guidance. Apart from the branding and labeling of subsidized or free products earlier reported, it was agreed that government agencies should conduct stricter stock inventory and control of those products.

### Capacity building

#### Knowledge gaps among service providers

Participants observed that training and the involvement of private sector providers in the RDT project increased the confidence of many primary stakeholders in service provision, and also in profits.

*“It has advanced our practice, we used to sell anti*-*malarial drug out indiscriminately once patients complain of malaria symptoms”.* (PPMV)

*“We didn’t know anything before but now we can stand in front of anybody and explain what we are doing”.* (PPMV)

Participants were willing to receive continuous training on the RDTs to further improve their confidence and consolidate their skills in carrying out the test procedure. There were calls to make subsequent trainings audience-friendly by explaining technical terms in simple language and to conduct trainings in local languages for the benefit of service providers domiciled in rural areas.

#### Limited number of participating private providers

It was reported that there was a high acceptance of RDTs by clients as it saved them time and effort that would have been spent going to a laboratory or a hospital and it also helped inform their choice of the drug to purchase. However, the participants acknowledged that the current numbers of enrolled private providers is a small fraction. The consensus was to train and involve more formal private facilities and PPMVs to achieve impact.

#### Limited number of qualified RDT trainers

The gaps reported in the providers’ capacities was related to the lack of an adequate number of trainers. To solve this and enable the enrolment of more service providers it was suggested that more RDT trainers should be recruited and trained.

## Discussion

Recent evidence [7] confirmed what has been known about the health-seeking behaviour of the majority of Nigerians; they often seek treatment and care for fever at private health establishments [3, 8]. This was the premise for the implementation of a pilot project on the deployment of quality-assured RDTs in the private sector between mid-2014 and mid-2016, with the support of the UNITAID. The interaction with a cross-section of stakeholders at the conclusion of the project provided valuable insights from their perspectives on important lessons that should be addressed in any effort to sustain or scale up services in the private sector.

This report showed that the consensus among stakeholders was that RDT uptake was widely acceptable by private providers and their clients in the three project sites. This represents an encouraging shift from earlier reports of a low uptake of RDTs in both public and private providers taken together [2]. A recent report by Mokuolu et al. [19] also substantiated this increasing interest to test before treatment among private providers in Nigeria. However, the encouraging outlook is not without problems, and not peculiar to only Nigeria [12], some of which were expressed by the participants at the concluding stakeholders’ meeting highlighted in this report.

Despite the identified challenges, there was strong agreement among the broad base of stakeholders that actions can be taken to ensure the sustainability and possibly scale up the use of RDTs for fever case management in the formal and informal private health sector. Operational challenges, such as a relatively low consumer-driven demand and slow stock depletion, should be addressed by increasing consumer awareness and provision of incentives. This serendipitously agreed with results of an interventional study reported by Aung et al. [20] in which price subsidy combined with intensified IEC increased uptake of RDTs among informal private providers in Myanmar. In a related study, the authors further showed that this incentive scheme proved to be more cost-effective relative to three other options [21].

Stakeholders suggested that decentralization by establishing product storage hubs will tackle the problem of delays in supply by distributors. Yeung et al. [22] had also emphasized the need for setting up “far-reaching distribution networks” to ensure availability of commodities in the least accessible areas for effective malaria case management in the private sector, owing to their peculiarities.

This report also highlighted the topical issue of adherence to RDT results by both providers and clients, which several other studies also identified as a sore point in the deployment of RDT, as an alternative to presumptive diagnosis when not recommended, and to microscopy when not feasible [19, 20, 23]. To address non-adherence, the meeting participants, largely made up of service providers, recommended training/re-training and closer supervision of providers by government and development partners; and in turn, counseling of clients by the providers. This submission of shared responsibility underscores the value of involving all levels of stakeholders in the decision-making tree [24].

Supportive supervision and capacity building also emerged as strong recommendations for improving on the poor data reporting, poor quality of service and the current small numbers of participating private providers. Hitherto, regulations in Nigeria had restricted PPMV from performing invasive procedures thus the scale of the concluded project, and of several other private sector RDT initiatives, minimally involved PPMVs. However, with the recent upturn of the restriction due to several advocacy efforts, the relevance of PPMVs in the deployment RDT for fever management in Nigeria’s huge private sector has soared.

The methodology employed in this report is a recognized limitation on how wide these findings could be generalized. However, the intention to demonstrate the value of engaging stakeholders in current and future health interventions was achieved, given the depth of information that the exercise generated.

## Conclusion

Exploring a systematic process of engaging a broad base of stakeholders when considering sustainability and scale-up plans for deployment of RDTs in the private sector yielded a wealth of information. Stakeholders reached a consensus on the feasibility of expanding the private sector RDT market by increasing demand-creation among consumers and training of providers; and integrating supportive supervision of private providers with existing public sector mechanism. It was also agreed that strengthening an efficient commodity supply system and establishing incentive mechanisms for providers/clients are essential to improve uptake of testing and retaining profitability to private providers.

## Abbreviations

ACT: artemisinin-based combination therapy
IEC: information, education and communication
RDT: rapid diagnosis test
M&E: monitoring and evaluation
PCT: Project Country Team
PPMV: proprietary patent medicine vendors
SOP: standard operating procedures
UK/DFID: UK Department for International Development
WHO: World Health Organization
